# First 'Rauisuchian' archosaur (Pseudosuchia, Loricata) for the Middle Triassic *Santacruzodon* Assemblage Zone (Santa Maria Supersequence), Rio Grande do Sul State, Brazil

**DOI:** 10.1371/journal.pone.0118563

**Published:** 2015-02-25

**Authors:** Marcel B. Lacerda, Cesar L. Schultz, Cristina Bertoni-Machado

**Affiliations:** 1 Instituto de Geociências, Laboratório de Paleovertebrados, Universidade Federal do Rio Grande do Sul, Porto Alegre, RS, Brazil; 2 Centro Superior de Tecnologia TECBrasil, Faculdade de Engenharias, Porto Alegre, RS, Brazil; University of Florence, ITALY

## Abstract

The ‘Rauisuchia’ are a group of Triassic pseudosuchian archosaurs that displayed a near worldwide distribution. In Brazil, their fossils are found only in the Santa Maria Formation (Paraná Basin) of the Rio Grande do Sul State, specifically in the Middle Triassic *Dinodontosaurus* assemblage zone (AZ) and the Late Triassic *Hyperodapedon* AZ (*Rauisuchus tiradentes*). Between these two cenozones is the *Santacruzodon* AZ (Middle Triassic), whose record was, until now, restricted to non-mammalian cynodonts and the proterochampsian *Chanaresuchus bonapartei*. Here we present the first occurrence of a rauisuchian archosaur for this cenozone, from the Schoenstatt outcrop, located near the city of Santa Cruz do Sul and propose a new species, based on biostratigraphical evidence and a comparative osteological analysis.

## Introduction

The ‘rauisuchians’ comprise a problematic group of Middle to Late Triassic pseudosuchian archosaurs that are traditionally bundled together due to shared similarities in cranial, pelvic and ankle morphologies, but may not represent a natural group [1–5]. As such, some workers proposed [3,6] that the name be used between commas to refer to them, independently if they are a monophyletic or not, while Rauisuchia is used to designate a monophyletic group [3–6]. This is the terminology applied in the present article. The last 20 years have seen an increase in the number of discoveries and redescriptions *e.g*. [7–19] along with recent cladistic analyses [4, 6, 19] which have contributed to a better understanding of the diversity and relations among ‘rauisuchians’ within Archosauria. Although the use of more taxa in recent phylogenies has contributed to a better understanding of the diversity found in these forms, their phylogenetic and taxonomic definitions still remain unclear and new studies and more complete specimens are needed to attempt to resolve many problematic topics [3, 4, 5, 6, 11].

**Fig 1:**
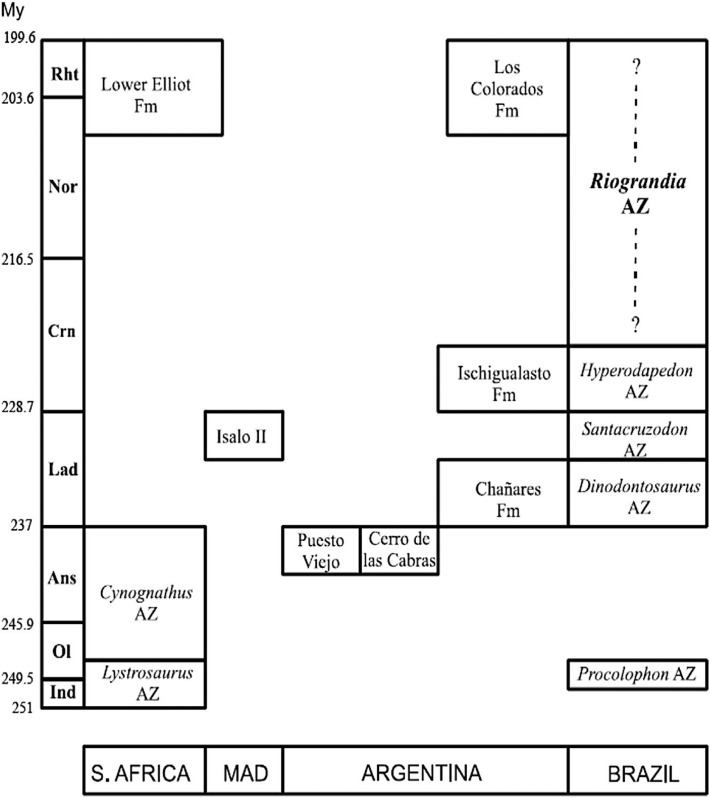
Comparative chart with the biostratigraphical framework of the Santa Maria Supersequence along with the ones from South Africa, Madagascar and Argentina. From [23].

‘Rauisuchians’ displayed a near worldwide distribution, with the exception of Oceania and Antarctica [3, 5, 20]. In Brazil, their fossils are found only in the Santa Maria Supersequence [21, 22], Paraná Basin, Rio Grande do Sul State, in the southern part of the country. This Supersequence encompasses two biostratigraphic units bearing ‘rauisuchians’; the Middle Triassic *Dinodontosaurus* Assemblage Zone (AZ) with the species *Prestosuchus chiniquensis*, ‘*Prestosuchus loricatus’* and *Decuriasuchus quartacolonia*, and the Late Triassic *Hyperodapedon* AZ, with the record of *Rauisuchus tiradentes* [5, 14, 23, 24]. Placed between these two biozones, occurs the *Santacruzodon* AZ, for which no ‘rauisuchians’ had been found until now. The faunal association of this biozone includes mainly traversodontid (*Santacruzodon hopsoni* [25]; *Menadon* sp. [26] and probainognathid (*cf. Probainognathus* [23]) cynodonts, the proterochampsian *Chanaresuchus bonapartei* Romer 1971 [27] and cranial fragments of dicynodonts [26, 27]. The presence of *Menadon* and the close taxonomic relationships between *Santacruzodon hopsoni* and *Dadadon isaloi* [28] indicates a temporal correlation between the *Santacruzodon* AZ and the Ladinian “Isalo II” fauna of Madagascar [28, 29] (Fig. 1).

In the present contribution, we describe the first occurrence of a ‘rauisuchian’ archosaur for the *Santacruzodon* AZ, based on an incomplete pelvic girdle, and discuss its taxonomic status, proposing it as a new species.

### Geological setting

**Fig 2:**
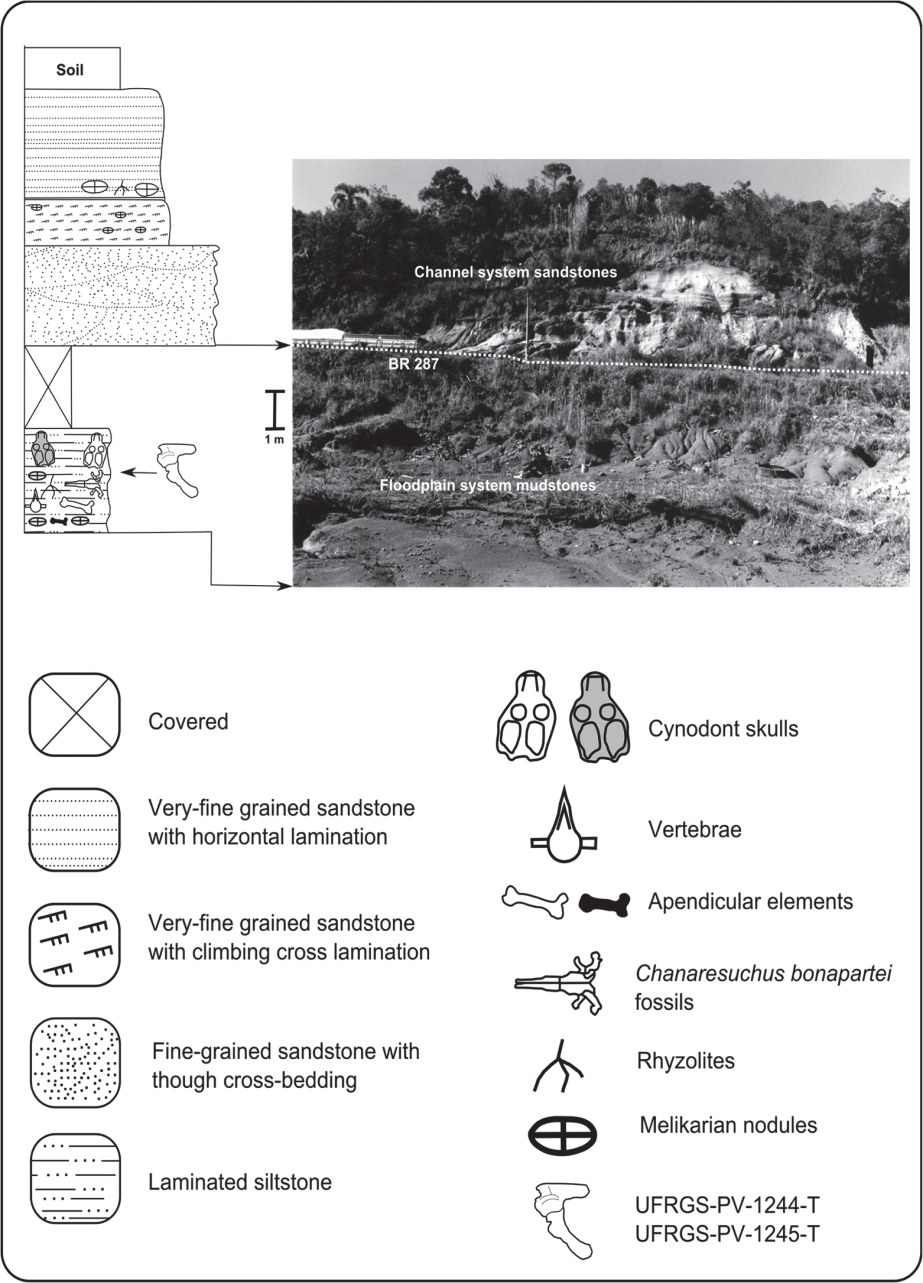
Stratigraphical profile of the upper portion of the Schoenstatt Outcrop (Modified from [31]).

The material here described came from the Schoenstatt site that is located on the outskirts of Santa Cruz do Sul city, (UTM SAD 69-22J-359794°E, 6709033°N) approximately 150km from the capital of Rio Grande do Sul State, Porto Alegre. It is a landfill that is exposed at the west margin of the RS-287 highway in the subdivision of Faxinal Velho, close to the Schoenstatt sanctuary [30]. The geological profile of the site shows an association of channel facies and floodplains (Fig. 2). The fossils occur in a level of massive red mudstones approximately 5 m thick that displays accumulations of disarticulated skeletal elements, with a predominance of skull and jaw elements, interpreted as a biogenic concentration formed by the accumulation of discarded remains by selective predators and carrion eaters [31].

## Materials and Methods

No special permits were required for the present study, which complied with all relevant legal regulations. The studied materials were collected and deposited in the paleovertebrate collection of the Laboratório de Paleontologia de Vertebrados of the Instituto de Geociências of the Universidade Federal do Rio Grande do Sul, which is a federal university and as such, specimen collection and housing complies fully with the laws that regulate fossil and mineralogical materials of Brazil. All the other relevant information, such as details of the site where the fossils were collected and their catalogue number are presented and discussed in the "Geological Setting" part of "Introduction" heading and in the present topic, respectively.

The specimen is represented only by an incomplete and disarticulated pelvic girdle, specifically a left ilium (UFRGS-PV-1244-T) along with a right and a left ischium (UFRGS-PV-1245-T). They were found disarticulated but closely associated. The similar dimensions of both ischia (the left one is 18.3cm long and the right one is 19.1cm) and the overall size and aspect of the acetabular area when the left ilium and ischium are placed in articulation suggest that all elements belong to the same individual. It was prepared using mechanical chisels, explorers and brushes.

### Systematic Paleontology

ARCHOSAURIA Cope, 1869 *sensu* Gauthier & Padian 1985

PSEUDOSUCHIA Zittel, 1887–1890 *sensu* Gauthier & Padian 1985

SUCHIA Krebs 1974 (*sensu* Benton & Clark 1988)

LORICATA Merrem 1820 (*sensu* Nesbitt 2011)

*Dagasuchus santacruzensis* sp. nov., gen. nov., urn:lsid:zoobank.org:act:A3B06825-1F56-4C95-8F75-4393AA32B752

Etymology: From the words *daga*, which is a large knife or dagger in the regional gaucho jargon, in reference to the pronounced iliac blade and *suchus* from the Greek word for crocodile; *santacruzensis* refers to the city of Santa Cruz do Sul, where the Schoenstatt outcrop is located.

Holotype: UFRGS-PV-1244-T, left ilium; UFRGS-PV-1245-T, right and left ischium, deposited in the paleontological collection of the Laboratório de Paleovertebrados of the Instituto de Geociências of the Universidade Federal do Rio Grande do Sul.

Horizon and Locality: Santa Maria Supersequence, *Santacruzodon* Assemblage Zone, Late Ladinian/Early Carnian, Schoenstatt outcrop, near Santa Cruz do Sul, Rio Grande do Sul State, Brazil.

Diagnosis: medium sized archosaur with imperforate acetabulum and preacetabular process of the ilium shorter than the postacetabular process, similar to that of Loricata (*sensu* Nesbitt, 2011), but differs from all known taxa within that grouping (*Saurosuchus galilei, Prestosuchus chiniquensis, Batrachotomus kupferzellensis*) and *Decuriasuchus quartacolonia* by the presence of the following combination of features: Ilium displays smooth dorsal and ventral margins of the iliac blade, articulation sites for two sacral vertebrae, a ‘plate-like’ ischium, with smooth anterodorsal area and a continuous anteroventral margin with no apparent notch.

### Nomenclatural Acts

The electronic edition of this article conforms to the requirements of the amended International Code of Zoological Nomenclature, and hence the new names contained herein are available under that Code from the electronic edition of this article. This published work and the nomenclatural acts it contains have been registered in ZooBank, the online registration system for the ICZN. The ZooBank LSIDs (Life Science Identifiers) can be resolved and the associated information viewed through any standard web browser by appending the LSID to the prefix “http://zoobank.org/”. The LSID for this publication is: urn:lsid:zoobank.org:pub:59314E74-4A8F-4077-A8F3-BAB5B3B99EB9. The electronic edition of this work was published in a journal with an ISSN, and has been archived and is available from the following digital repositories: PubMed Central and LOCKSS.

## Description

**Fig 3:**
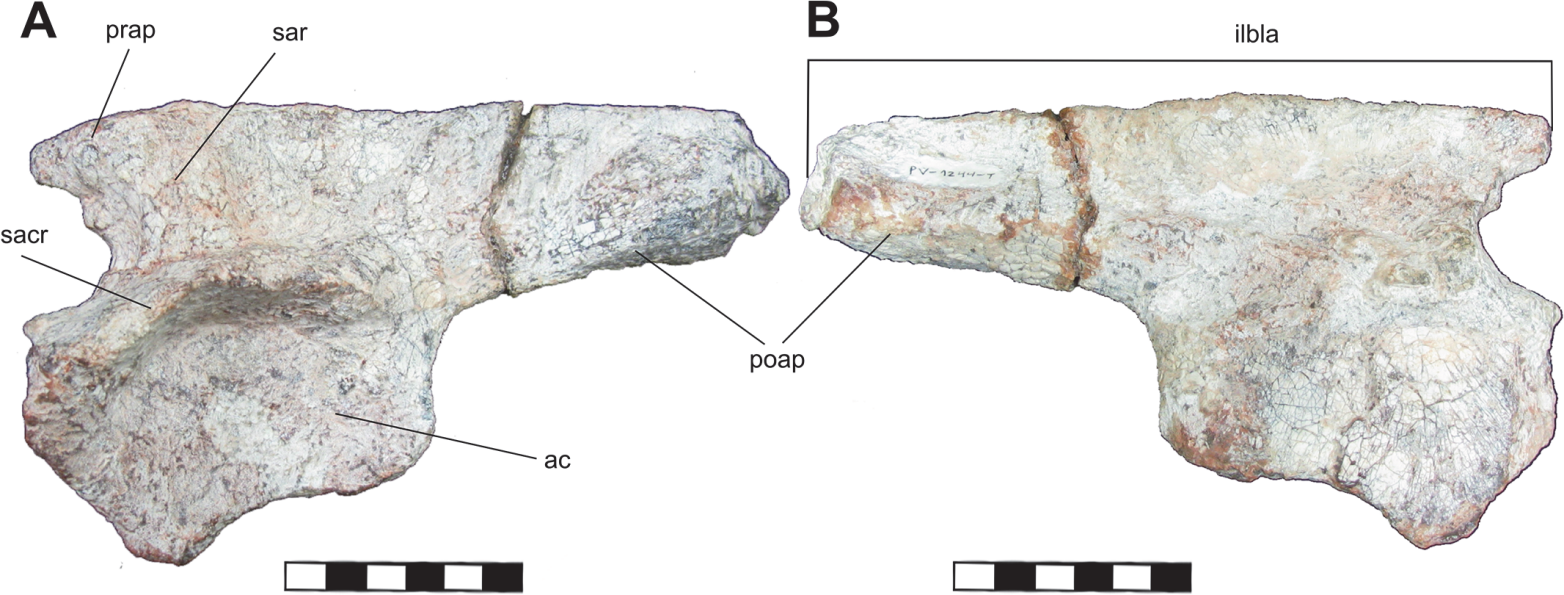
Left ilium (UFRGS-PV-1244-T) in lateral (A) and medial (B) views. Abbreviations: **ac,** acetabule; **ilba**, iliac blade; **prap**, pre-acetabular process; **poap**, post-acetabular process; **sar**, supra-acetabular crest; **sacr,** supra-acetabular ridge. Scale: 5cm.

The ilium is well preserved, with roughly 19 cm in length and 10 cm tall (Fig. 3). The contact of the blade with the acetabular area is continuous, not displaying a waisted region between both areas. The former is overall complete, with what appears to be only minor damage on both extremities. The dorsal margin of the iliac blade, in lateral view, is smooth and straight, with no sign of any rugose surface as those described for *Batrachotomus* and *Prestosuchus*. Perpendicular to its longitudinal axis there is a low (but distinct) antero-posteriorlly wide ridge that projects antero-dorsally on its lateral area from the dorsal border of a robust supra-acetabular crest, dividing the iliac blade into a short preacetabular and longer postacetabular process. The former has the same length as the anterior margin of the acetabular region, with the area near the anterior margin of the crest being preserved, while the postacetabular process projects antero-posteriorlly into a dorso-ventrally tall and compressed blade of bone that slightly tapers posteriorly. The ventroposterior portion of this blade is gently expanded laterally, which occupies the rest of the posterior tip

In dorsal view, the blade is sinuous, with a laterally concave anterior area where the supra-acetabular ridge is located.

The ventral portion of the acetabulum, in lateral view, especially close to the area of articulation with the ischium and the pubis, was lightly eroded by weathering and when the ilium and the ischium are placed in articulation the contact is extremely narrow, indicating that the acetabulum is most likely imperforate. The acetabulum is overall slightly oval and deep, gradually rising laterally up to the anterior portion of the supra-acetabular rim.

In medial view, the anterior portion of the base of the iliac blade displays a medially expanded surface with a dorsal depression that would be the site for the articulation of the rib of the first sacral vertebrae. Posteriorly to this area, along the blade, there is a shallow depression along its length, up onto a medially projecting area of the base of the blade, at about 45° to the sagittal plane, which posteriorly forms a platform for the articulation of the rib of the second sacral vertebrae. The ventroposterior portion of the blade, between the medial crest and the lateroventral margin of post-acetabular process does not display any fossa or depression. On the acetabular area, there is a slightly oval sub-horizontal depression located where the abovementioned platform begins, which would correspond to another rib articulation site.

**Fig 4:**
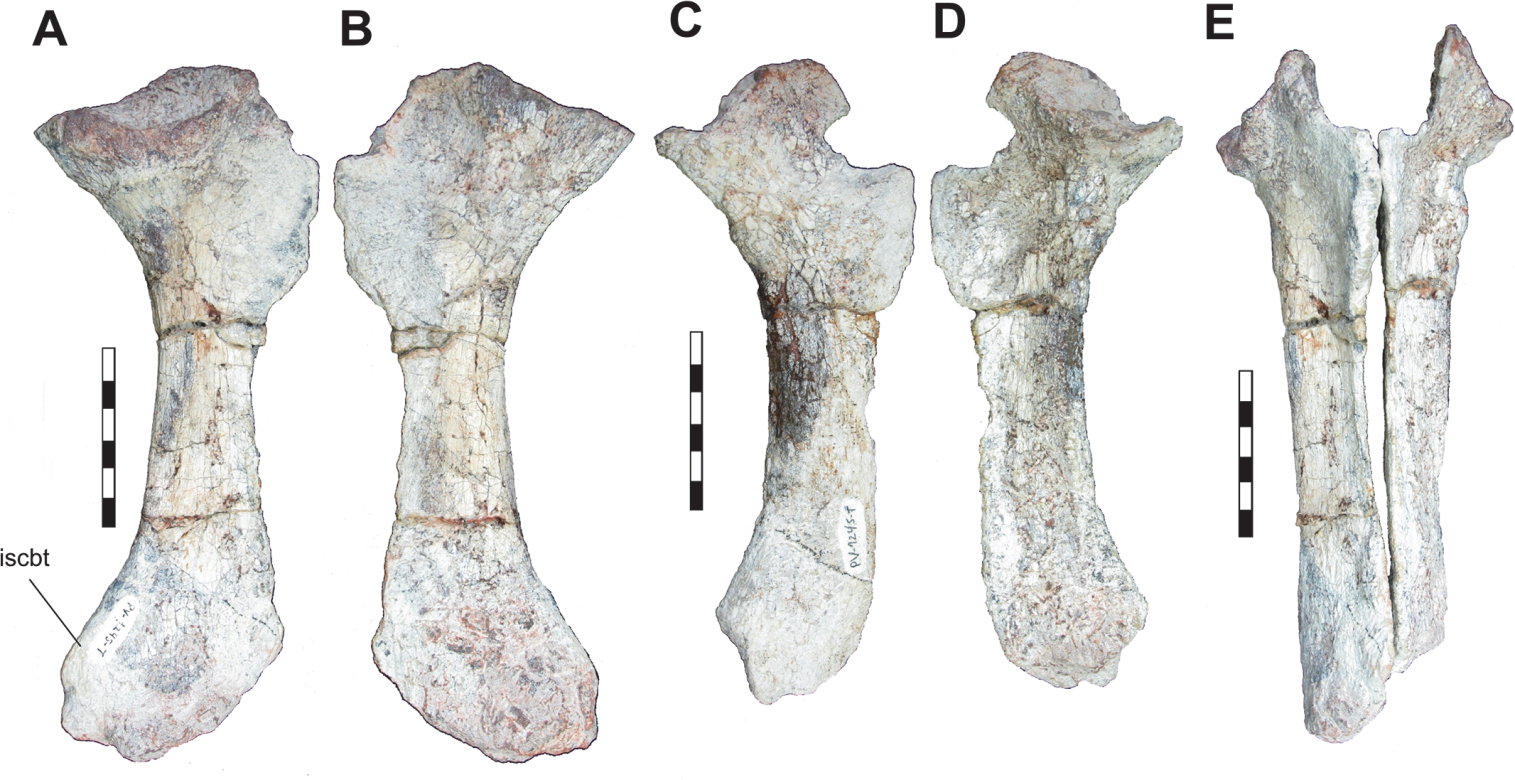
Right ischium in lateral (A) and medial (B) views; Left ischium in medial (C) and lateral (D) views; Both ischia in articulation in anterior view (E). Abbreviations: **iscbt**, Ischium boot. Scale: 5cm.

The right ischium is better preserved than the left one (Fig. 4). Both are dorso-ventrally elongated plate-like elements, with a more robust and smooth dorsal body and expanded proximal and distal ends. In the right ischium, the proximal area, in lateral view, displays a sinuous dorsal portion, and is bordered ventrally by a laterally expanded semi-circular ventroposterior border of the acetabulum. Posteroventrally to this area, a sheet of bone projects ventrally and expands to form an irregular lateromedially compressed surface that is more distinct on the first third of the ischium, that decreases in size posteriorly and continues along the body of the bone up to distal end. The distal ends of the ischia, in lateral view, display an expanded and posteriorly directed “ischium boot”.

The ventral margins of both elements are partially eroded by weathering. If both elements are positioned in relative articulation, the lateromedially compressed area is the main surface for the articulation of both ischia anteriorly. Distally, a gap appears between them in anterior view, but it is unclear if this gap is natural or a result of diagenetic alteration to the bones. The case for the latter is reinforced by the presence of some distortion in the left ischium, which is more transversally curved than the right one.

Additionally, there is a small, roughly triangular-shaped fragment of bone that was preserved on the medial area of the ilium. However, this piece does not display any diagnostic feature and thereby is not figured here.

## Discussion

The studied pelvic girdle presents an imperforate acetabulum, a laterally expanded, semicircular supra-acetabular crest and an antero-dorsally orientated supra-acetabular ridge. These features, as well as the overall aspect and the antero-posterior length of the iliac blade match those described for ‘rauisuchian’ taxa within Loricata *sensu* Nesbitt (2011), differing from those of other more derived forms within Poposauridea. The comparative osteological analysis indicates that it is morphologically similar to *Saurosuchus galilei* [32] of the Ischigualasto Formation (Carnian), *Batrachotomus kupferzellensis* [13] of the Lettenkeuper of Germany (Late Ladinian), as well as *Prestosuchus chiniquensis* [24] and *Decuriasuchus quartacolonia* [14] of the *Dinodontosaurus* AZ (Ladinian) of Brazil. The the overall aspect of the ilium is different from one originally described for *Rauisuchus tiradentes* by Huene [33] (BSPG AS XXV 88), although now considered as not belonging to the same individual as the lectotype based on size differences [34], the acetabule is restricted to the ilium, while in the presently described specimen it is likely composed by all three pelvic elements. Although it is similar to *Stagonosuchus nyassicus* from the Manda Formation (Anisian) it differs in having a significantly more pronounced supra-acetabular crest, a supra-acetabular ridge and absence of a medial protuberance on the lateral surface of the iliac blade [35, 36].

**Fig 5:**
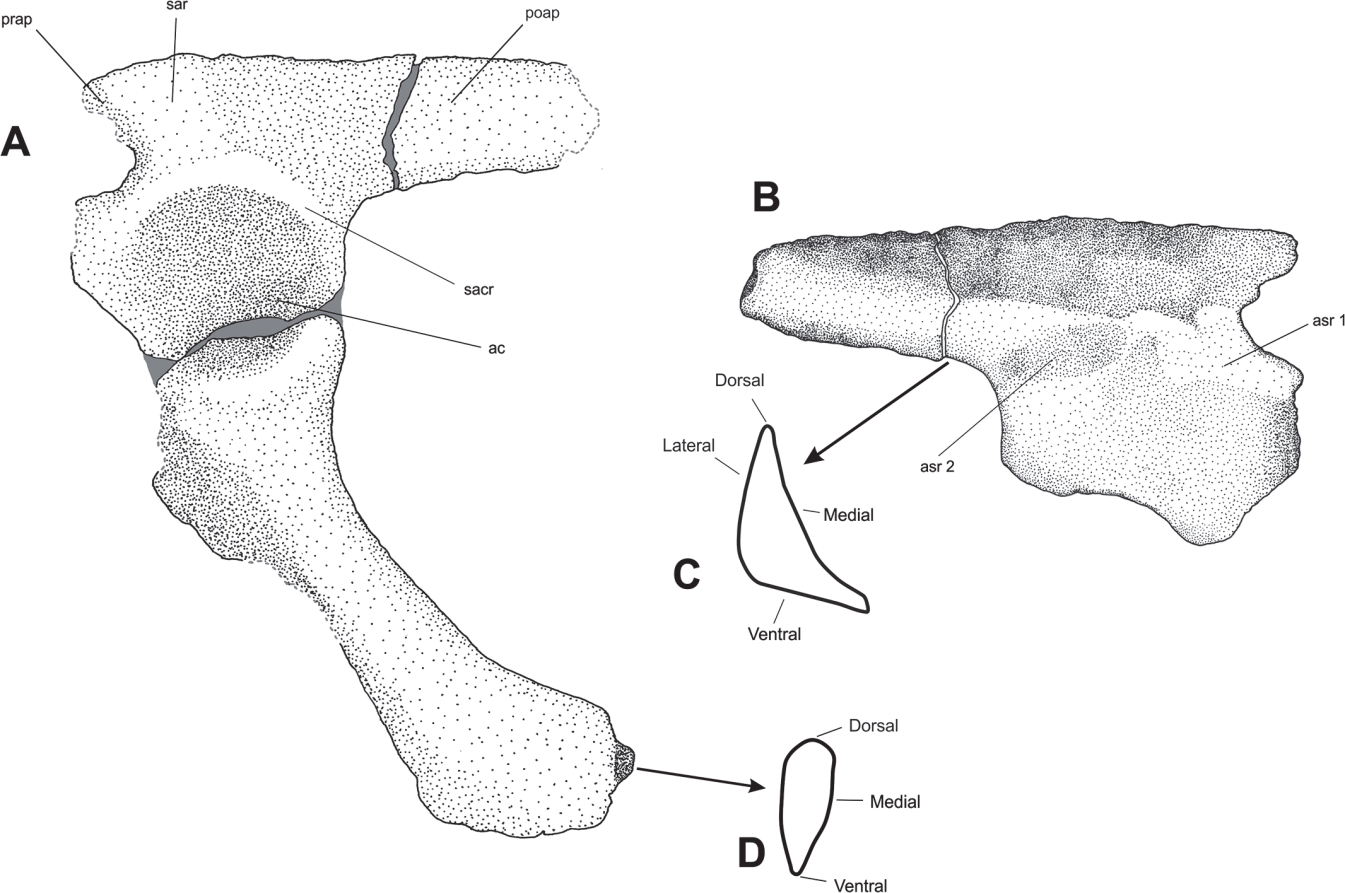
(A) Composite illustration of the left ilium with thee better preserved right ischium in articulation; (B) illustration of the ilium in medial view; (C) cross-section of the ilium in posterior view; (D) cross-section of the ischium in distal view. Abbreviations: as Fig. 2 plus: **asr 1**, articulation site for the sacral rib of the first sacral vertebra; **asr 2,** articulation site for the sacral rib of the second sacral vertebra. Damaged areas in grey.

Additional diagnostic features would be the articulation sites for only two sacral vertebrae (Fig. 5), a condition that is described for *Prestosuchus chiniquensis* [24, 37], *Saurosuchus galilei* [34] and *Decuriasuchus quartacolonia* [14] but not for *Batrachotomus kupferzellensis*, which has 3 [13].

The pelvic girdle here described differs from that of *Decuriasuchus quartacolonia* [14] by an overall larger acetabulum, shorter iliac blade and a more sinuous and plate-like ischia. Additionally, in the specimen MCN PV10105c figured in França et *al*. [14], the ilia are comparatively smaller and more gracile than the one here described (MBL pers. obs.). These features might be considered problematic in differentiating species, since it is also possible to attribute such variations to intraspecific factors, however, as França et *al*. [14] considered all specimens of *Decuriasuchus quartacolonia* as adult forms due to the presence of closed sutures between the skull elements as well as ossified vertebrae, the overall larger size of the present specimen can be used to differentiate it from that taxon.

Another pelvic girdle attributed to *Prestosuchus chiniquensis* by Nesbitt [4]-specimen UFRGS-PV-0152-T—displays similar dimensions and overall aspect of the one here described, but differs in the presence of an iliac blade which is much shorter dorsoventrally and that displays rugosities along its anterodorsal surface along with an ischium with a marked “notch” on the anteroventral margin. As this specimen is currently being studied as the subject of a PhD thesis, it is not figured here aside from the former comments.

The ischium differs from *Prestosuchus chiniquensis* [24, 33, 37] and *Saurosuchus galilei* [32] due to a more plate-like than rod-like morphology ([4]: character 9) and the absence of ridges on the dorsal surface of the iliac blade. It is possible to consider this feature as not morphologically significant, since the comparatively smaller size of the specimen in regards the above-mentioned species could be considered as ontogenetic and such ridges should be more developed as the animal grew, but this appears to be not the case, since the material is well preserved and no ridges are apparent. Additionally, the ischia further differs from *Prestosuchus chiniquensis* due to the absence of a notch on the ventral margin ([4]: character 296) and displaying a smooth anterodorsal surface, distally to the expanded proximal area, which lacks a low dorsal crest that is considered a sinapomorphy for this species, being present in the lectotype of *Prestosuchus chiniquensis* described by Huene [33, 37] and assigned by Krebs [38], with a more pronounced one in the more complete specimen (UFRGS-PV-0629-T) attributed to this taxon (see discussion in [24]). This comparative morphological analysis shows that none of the previously described species share the combination of features that is found in the presently described specimen. As such, it is clearly distinct from other ‘rauisuchians’, but the absence of clear autapomorphies combined with the incompleteness of the individual makes the proposition of a new taxon questionable.

**Fig 6:**
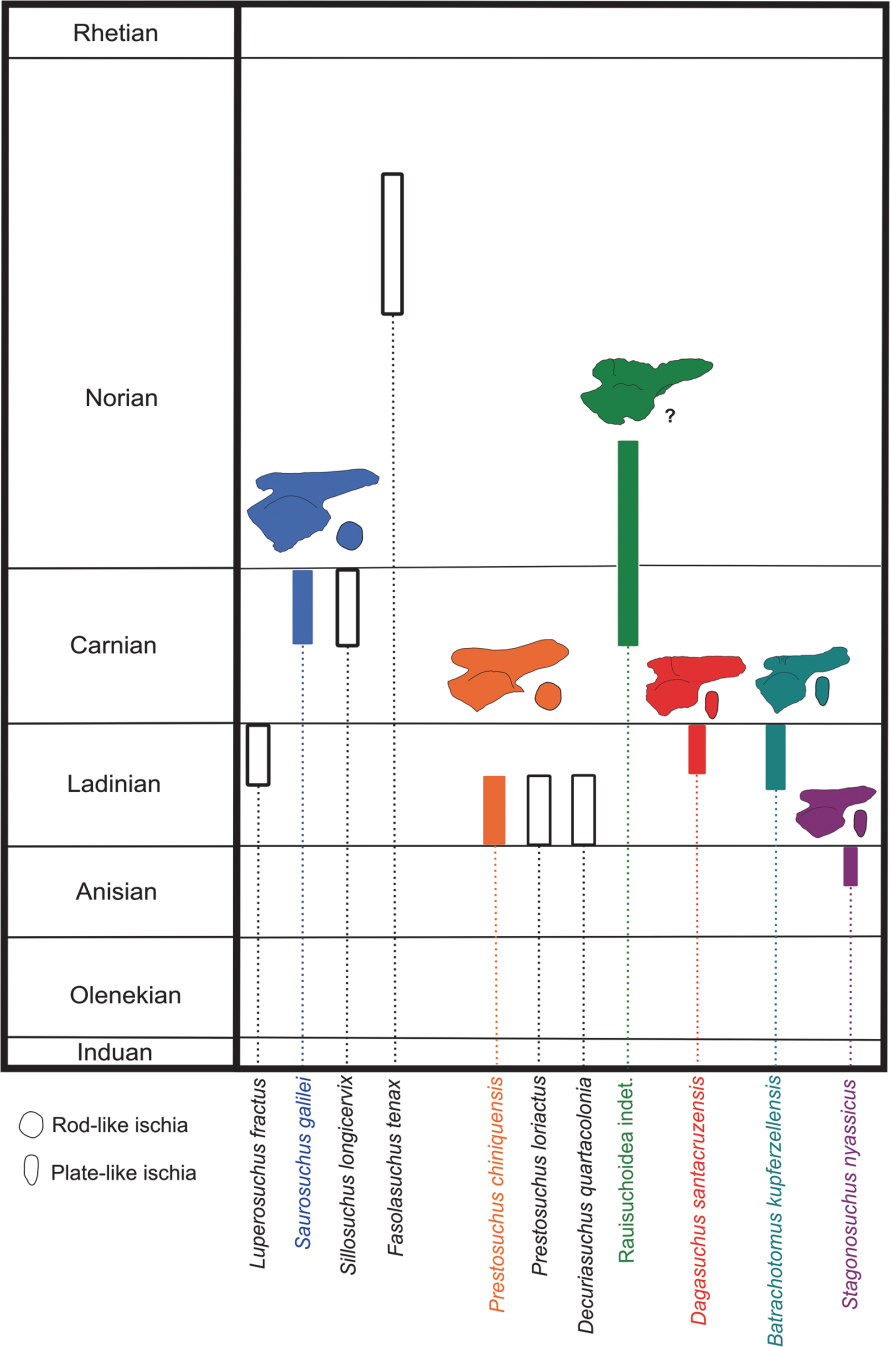
Temporal distribution of ‘rauisuchian’ taxa of South America and those with morphology of ilia and ischia similar to *Dagasuchus santacruzensis*, emphasizing the shape of the supra-acetabular crest and supra-acetabular ridge on the ilium and the outline of the section of the ischium: *Stagonosuchus nyassicus* (A) and *Batrachotomus kupeferzellensis* (B) based on [3]; *Prestosuchus chiniquensis* (C) based on [24] (2010); Rauisuchoidea indet. (**D**) based on [34]; *Saurosuchus galilei* based on [32]. On the right, comparative temporal distribution of these species, along with other South American ones (Modified from [5]). Comparative bones are not to scale.

Additional evidence to support a new taxon would be the biostratigraphical position of this specimen in the Santa Maria Supersequence. As presented earlier, all other ‘rauisuchian’ taxa have been limited to the *Dinodontosaurus* and *Hyperodapedon* assemblage zones. The *Santacruzodon* AZ is a unique third order sequence within the Santa Maria Supersequence, with paleofaunal correlation with the “Isalo II” fauna from Madagascar [22, 23, 25, 26], where no ‘rauisuchian’ has been described. However, the presence of *Chanaresuchus* [27, 30] in both the *Dinodontosaurus* and *Santacruzodon* assemblage zones, as well as in the Chañares and Ischigualasto faunas would indicate that at least one taxon continued across the Ladinian/Carnian transition. As such, the possibility of a previously described Ladinan ‘rauisuchian’ taxon that existed up to the Late Ladinian/ Early Carnian cannot be completely excluded. However, we consider that all the other evidences presented here (Fig. 6) along with the biostratigrafical data justifies the distinction of this specimen as unique among other ‘rauisichian’ taxa, and as such, would be more than only a “rauisuchian indet.” or a similar provisional designation.

## Conclusion

Although incomplete, the combination of features of the described specimen permits a taxonomic designation to a new ‘rauisuchian’ species *Dagasuchus santacruzensis*. This new species adds to the knowledge of the diversity of the group in the Triassic and fills in a gap in the temporal distribution of these forms in South America. Major steps in the study of these forms have been made in recent years, however, new finds are still required to clarify the problematic topics presented in this paper, in ‘rauisuchian’ studies and Triassic continental biostratigraphical data in general.
